# A Review of the Consequences of Gut Microbiota in Neurodegenerative Disorders and Aging

**DOI:** 10.3390/brainsci14121224

**Published:** 2024-12-03

**Authors:** Amanda A. Menezes, Zahoor A. Shah

**Affiliations:** Department of Medicinal and Biological Chemistry, College of Pharmacy and Pharmaceutical Sciences, The University of Toledo, Toledo, OH 43614, USA; amanda.menezes@rockets.utoledo.edu

**Keywords:** cognitive decline, neurodegenerative diseases (NDDs), Alzheimer’s disease, gut microbiota, gut–brain axis, neuroinflammation, fecal microbiota transplantation, probiotics, prebiotics, microbial therapies

## Abstract

Age-associated alterations in the brain lead to cognitive deterioration and neurodegenerative disorders (NDDs). This review with a particular focus on Alzheimer’s disease (AD), emphasizes the burgeoning significance of the gut microbiota (GMB) in neuroinflammation and its impact on the gut–brain axis (GBA), a communication conduit between the gut and the central nervous system (CNS). Changes in the gut microbiome, including diminished microbial diversity and the prevalence of pro-inflammatory bacteria, are associated with AD pathogenesis. Promising therapies, such as fecal microbiota transplantation (FMT), probiotics, and prebiotics, may restore gut health and enhance cognitive performance. Clinical data remain insufficient, necessitating further research to elucidate causes, enhance therapy, and consider individual variances. This integrative approach may yield innovative therapies aimed at the GMB to improve cognitive function and brain health in older people.

## 1. Introduction

Physiological changes in the brain due to aging can result in a gradual decline in overall brain health and cognitive function. The deterioration in brain function in age-related diseases like Alzheimer’s disease (AD) lowers the patients’ quality of life. AD is characterized by senile plaques of amyloid beta (Aβ) peptides, neurofibrillary tangles of hyperphosphorylated tau protein linked to microtubules, and neuroinflammation leading to neurodegeneration [1].

**Figure 1:**
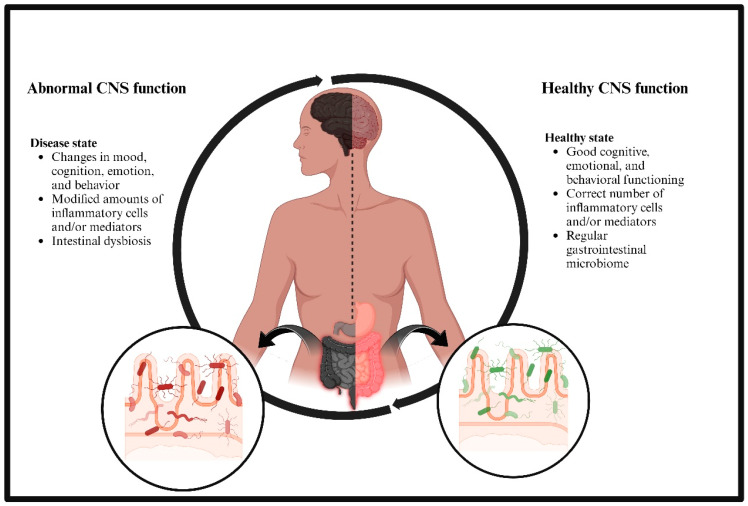
Relationship between gut microbiome and central nervous system functioning in neurological disorders. Created with BioRender.com.

**Figure 2:**
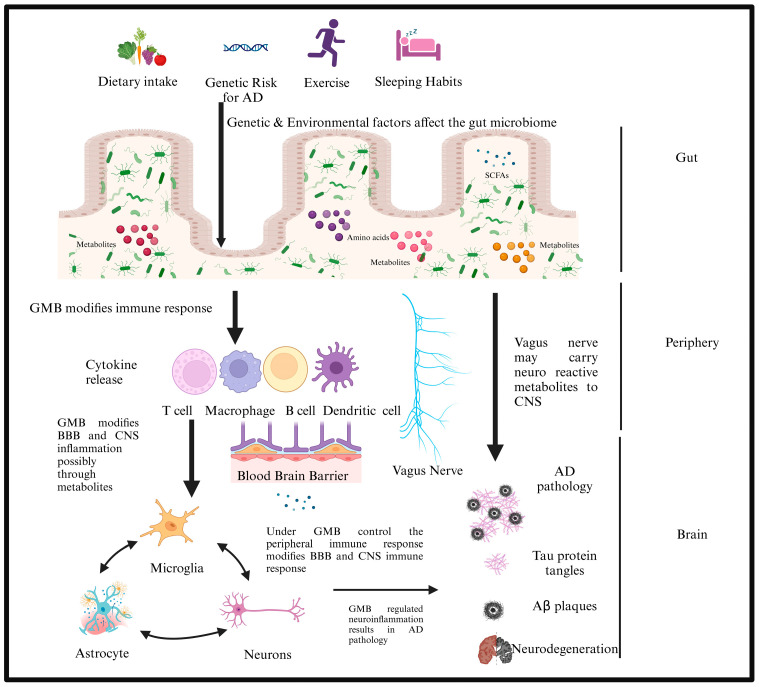
The hypothesis posits that environmental factors such as nutrition, sleep, and exercise, along with genetic predispositions, contribute to an inflammatory milieu inside the gut microbiota (GBM), resulting in alterations in composition and diversity over time. These alterations affect metabolites originating from the GMB and peripheral immunity, modifying the blood-brain barrier (BBB) and central nervous system (CNS) cell types, potentially influencing AD. Created with BioRender.com.

The gut microbiota (GMB) comprises various microorganisms, including bacteria, archaea, protozoa, viruses, and fungi. Research indicates that the GMB may play a role in regulating neuroinflammation in various neurological disorders, including multiple sclerosis (MS) [2], Parkinson’s disease (PD) [3,4], and AD [5,6,7,8]. The regulation of neuroinflammation by GMB can come about via both direct and indirect mechanisms, as seen in Figure 1. Alterations in the GMB may influence the synthesis of metabolites by bacteria and the immune response in the peripheral system. This may subsequently affect the immune response in the central nervous system (CNS) during neurological disorders (Figure 2) [9]. Recent studies demonstrate that individuals with AD possess altered GMB [9] in contrast to those without AD [10,11]. Furthermore, investigations utilizing mouse models of AD have demonstrated that altering the gut microbiome might influence the progression of pathology and neuroinflammation [5,6,7,8,10,11,12]. The GMB significantly influences the production of neurotransmitters such as serotonin and dopamine, primarily through metabolic pathways and bacterial interactions with the host’s enteric nervous system.

Certain gut bacteria influence serotonin synthesis by metabolizing tryptophan, a precursor of serotonin. For instance, species like *Escherichia coli* and *Bifidobacterium* increase the availability of tryptophan, promoting its conversion into serotonin in the gut’s enterochromaffin cells. Additionally, short-chain fatty acids (SCFAs) from bacteria like *Bacteroides* and *Clostridium* stimulate tryptophan hydroxylase (TPH1), the key enzyme for serotonin synthesis in the gut. However, gut-derived serotonin does not cross the blood-brain barrier but modulates brain function through the gut–brain axis (GBA) via vagus nerve signaling and immune modulation [13]. Some bacterial species, such as *Enterococcus faecalis* and *Bacillus* spp., produce dopamine by converting L-DOPA into dopamine through bacterial decarboxylase enzymes. This dopamine can influence the gut’s enteric nervous system and impact systemic neurotransmitter levels, potentially affecting mood and behavior [14]. Gut-derived neurotransmitters indirectly impact the central nervous system (CNS) through signals from the GMB that activate the vagus nerve, influencing brain regions related to mood and cognition. GMB can produce metabolites like SCFAs, influencing neuroinflammation and the blood-brain barrier (BBB)’s integrity, thus affecting neurotransmitter regulation in the brain [13].

This review aims to summarize the latest research on the role of GBA in neurodegenerative diseases (NDDs), with a key focus on age-related conditions such as AD, and to elucidate this communication pathway by focusing on its key components, mechanisms, and potential therapeutic approaches.

## 2. The Gut–Brain Axis

The GBA signifies the bidirectional connection between the gut bacteria and the brain. The neurological, immunological, and endocrine systems collaborate to enable this intricate link (Figure 2). It is crucial for regulating mental and physical health and maintaining homeostasis in the microbiota, CNS, and gastrointestinal tract systems. This modulation occurs via chemical messengers’ direct or indirect influence, such as microbial hormones and metabolites [15,16,17]. The key features of the GBA and its potential therapeutic benefits for neurodegenerative diseases remain inadequately defined. The gut and brain interact through two distinct neuroanatomical pathways.

### 2.1. Nervous System Routes for Gut–Brain Axis Communication

**Figure 3:**
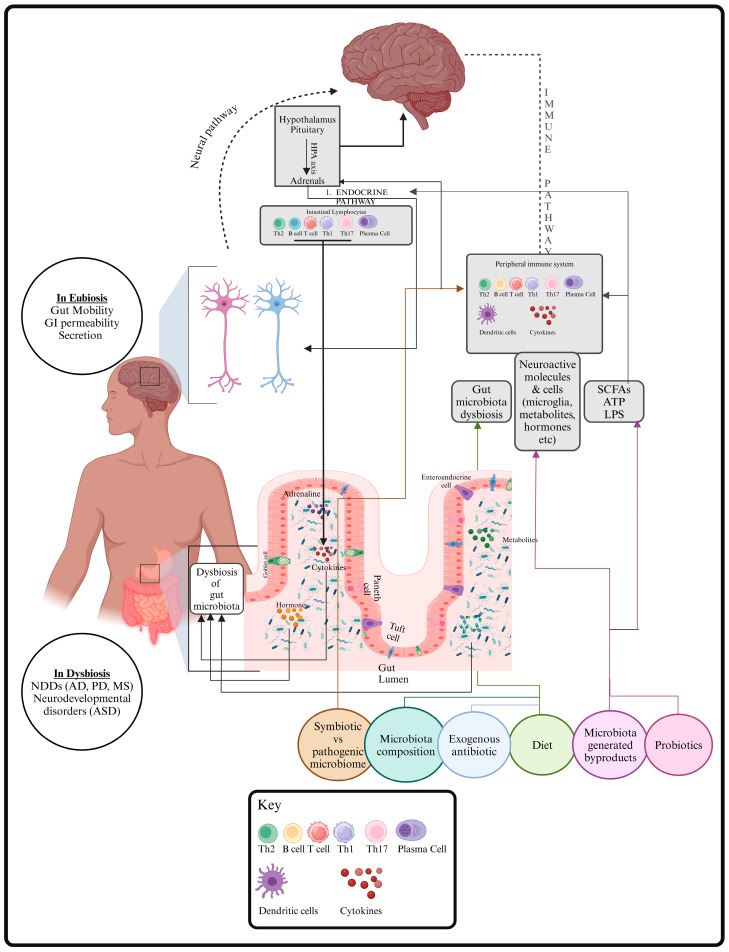
Mechanisms for bidirectional communication in the gut–brain axis (GBA). Created with BioRender.com.

The vagus nerve and the autonomic nervous system of the spinal cord facilitate direct communication between the brain and the stomach. The bilateral interaction between the gut and brain is facilitated by the biocommunication of the enteric nervous system (ENS) within the gastrointestinal tract, which is also influenced by the autonomic nervous system and the vagus nerve [18]. Bacteria create a direct neurological connection between the brain and the gastrointestinal microbiota by stimulating the enteric nervous system afferent neurons and utilizing the vagus nerve [19]. Vagal activation is linked to various positive effects on the GMB and the probiotics resulting from vagal activity, including anti-inflammatory benefits [18]. Multiple preclinical investigations have shown that the pathophysiology and pathogenesis of intestinal disorders, including inflammatory bowel disease (IBD) and irritable bowel syndrome, along with neurological and psychiatric conditions such as anxiety, depression, AD, MS, and PD, are linked to an imbalance in gut microbial communities, termed “dysbiosis” in the GMB (Figure 3) [20,21,22,23,24,25,26,27].

### 2.2. Gut Microbiota and Neurodegenerative Disorders

A healthy gut microbiome is characterized by a balanced and diverse community of microbes, primarily from the following phyla: *Firmicutes*, *Bacteroidetes*, *Actinobacteria*, and *Proteobacteria*, which represent significant phyla within the domain of bacteria. 

Beneficial bacteria contribute to nutrient metabolism by synthesizing vitamins, specifically B vitamins and vitamin K, and by promoting the fermentation of dietary fibers into SCFAs, including butyrate, propionate, and acetate. SCFAs are essential for energy homeostasis, the preservation of gut barrier integrity, and the regulation of immune responses [28,29]. Gut bacteria are crucial in regulating innate and adaptive immune systems. *Lactobacillus* and *Bifidobacterium* contribute to an anti-inflammatory environment by interacting with dendritic and T-regulatory cells [30]. Dysbiosis is an imbalance in microbial composition characterized by a loss of beneficial bacteria and an overgrowth of pathogenic species. This imbalance is associated with various conditions: IBD is characterized by diminished levels of *Faecalibacterium prausnitzii*, a significant butyrate producer, which correlates with heightened inflammation in Crohn’s disease [29]. Neurodegenerative disorders are associated with changes in GMB composition, which may influence conditions such as Parkinson’s and Alzheimer’s via the microbiome-GBA. Dysbiosis can result in heightened gut permeability, often referred to as “leaky gut”, which permits detrimental substances to enter systemic circulation and provoke neuroinflammation [31]. Fecal microbiota transplantation (FMT) is a procedure that entails the transfer of fecal matter from a healthy donor into a patient’s gastrointestinal system to reestablish microbial equilibrium. This treatment is mainly utilized for *Clostridium difficile* infections (CDI) and is currently being investigated for additional conditions; the mechanism of action involves FMT’s restoration of beneficial bacterial populations, which in turn re-establishes metabolic functions and immune regulation. For instance, it can enhance the production of SCFAs, which possess anti-inflammatory properties [29]. Recent research indicates that FMT may reduce the symptoms of neurodegenerative diseases through the modulation of the gut–brain axis. In PD models, FMT enhanced motor functions and decreased neuroinflammation [29].

AD is a pervasive and irreversible neurological disorder marked by progressive neuronal degeneration, leading to deficits in memory and cognitive abilities. It is the most common form of dementia among the elderly population [32]. Individuals with AD demonstrate considerable impairments in memory, behavior, and learning, which affect their daily activities. In AD, the abnormal phosphorylation of the microtubule-associated protein tau in the dendrites and axons of cortical neurons leads to aggregation, resulting in Aβ deposition around or outside neurons and subsequent progressive synaptic failure [3,33,34]. Neuronal death is a consequence of Aβ accumulation and tau protein aggregation, which lead to decreased microtubule stabilization, synaptic failure, and the disruption of Ca^2+^ balance in neurons [35,36]. The core mechanisms underlying AD are still not fully understood despite extensive research into its etiopathology, and current Aβ therapies provide only limited symptom relief [37].

Evidence suggests that AD may have a microbial origin, although this theory is not new [38]. The diseases’ biomarkers, including tau/Aβ42 and phosphorylated tau, are associated with metabolites derived from the GMB present in the cerebrospinal fluid of AD patients. This indicates that GMB may play a role in the onset of AD [35]. A study revealed significant differences in the gut microbial communities between wild-type mouse model controls and Aβ precursor protein transgenic mice (APP) after performing bacterial 16S rRNA sequencing analysis on mouse fecal samples [11]. Transgenic mouse models displaying characteristics of AD have been shown to possess a variety of gut bacteria, and the effects of microbial intervention on AD have been explored [8,11]. Additionally, studies involving germ-free (GF) mice indicated that the lack of microorganisms resulted in the absence of neuroinflammation and amyloid plaques [11].

Fecal samples from AD patients exhibited significantly greater abundance of two bacterial taxa—*Escherichia* and *Shigella*, associated with inflammatory responses—when compared to those from healthy controls, as indicated by cross-sectional studies [39]. Individuals exhibiting cognitive impairment and brain amyloidosis may experience two key factors linked to a peripheral inflammatory condition: a reduction in the prevalence of anti-inflammatory *Eubacterium rectale*, and an elevation in pro-inflammatory *Escherichia* and *Shigella* bacteria. Dysbiosis of the GMB and systemic inflammation are posited to be interconnected, potentially contributing to the neurodegeneration observed in patients with AD. The results stem from limited research, indicating that further studies with larger statistical samples are necessary to evaluate the influence of GMB on the progression of AD.

A recent study indicated that the application of synthetic neurotoxic inhibitors for the treatment of AD yielded positive outcomes; *Porphyromonas gingivalis*, a bacterium associated with chronic periodontitis, was detected in the brains of AD patients within this research [40]. The colonization of the brain by these bacteria led to an increase in Aβ1-42 production. Additionally, the tau protein Aβ1-42 is adversely affected by the neurotoxic gingipains [39].

Shukla et al. [40,41] identified a possible association between the dysbiosis of GMB and neuroinflammation related to AD. The activation of the peripheral inflammasome, which exacerbates neuroinflammation during the progression of AD, was positively correlated with the increased expression of gut NLRP3. Consequently, notable alterations were observed in the GMB composition of 5xFAD mouse models at various ages when compared to age-matched control mice. Additionally, 5xFAD mice exhibited impaired gut barrier function, evidenced by the reduction of adherens and tight junction proteins compared to their non-transgenic counterparts. Moreover, the increased expression of proteins associated with the gut microbial inflammasome may serve as a critical initiator for activating subsequent inflammatory and cytotoxic mediators. Consequently, gastrointestinal NLRP3 may activate NLRP3 inflammasome-mediated neuroinflammation. Thus, in individuals with genetic predispositions, the manipulation of GMB may represent a viable therapeutic strategy for neurological disorders associated with AD [42]. Furthermore, Wang et al. [42] investigated the gastrointestinal Aβ load prior to its impact on the brain, and its involvement in GBA interaction and AD. The researchers found that absorption dysregulation preceded cerebral aggregation, with vascular Aβ peptide deposition in the intestinal epithelial barrier (IEB) impairing the IEB in Tg2576 mouse models of AD, both in presymptomatic and symptomatic stages, compared to wild-type models. The study found that changes in the GBA are associated with increased levels of IP-10, vascular endothelial growth factor (VEGF), and IL-9, which are three inflammatory plasma cytokines.

Wang et al. developed a novel technique utilizing GBA to explore the mechanisms underlying Aβ pathology in AD and to translate GMB manipulation into therapeutic applications. Wang et al. gathered fecal pellets for further analysis from APPSWE/PS1DE9 mice that underwent fecal donor transplantation from aged (16 months) APPSWE/PS1DE9 mice following a brief antibiotic cocktail treatment. The main factor contributing to FMT reconstitution in mice treated with antibiotics prior to intervention was the presence of donor sources such as *Coriobacteriaceae* and *Clostridium*, leading to the enhanced plaque deposition of antibodies. Activation of the astrocytes surrounding Aβ plaques was notably diminished following microbiota engraftment, in contrast to microglia [42]. In animal models of AD, prolonged administration of broad-spectrum antibiotics may decrease antibody accumulation and modulate innate immune responses that influence antibody-related amyloidosis [5]. Additionally, regular administration of antibiotic cocktails to transgenic mice decreased the aggregation of microglia and astrocytes around amyloid plaques in the hippocampal regions, along with insoluble Aβ plaques [6,8]. A notable reduction in *Butyricicoccus* and *Ruminococcus*, alongside an increase in *Proteobacteria* and *Verrucomicrobia*, was observed in mice exhibiting AD phenotypes. This was determined through a comparison of the fecal SCFAs and microbial composition between wild-type and AD mouse models across various ages. The results demonstrate changes in microbial diversity and composition. The decrease in SCFA levels suggests a disturbance in at least 30 metabolic pathways [43]. A prior study indicated that increased Aβ accumulation contributes to the pathophysiology of AD, while microglial activation hinders the removal and degradation of Aβ. Moreover, elevated Aβ deposits stimulate the release of pro-inflammatory mediators from microglia, such as nuclear factor kappa B (NF-κB), reactive oxygen species, inducible nitric oxide synthase, and cyclooxygenase-2, subsequently leading to neuroinflammation in individuals with AD [44]. The findings indicate that particular species of GMB may activate Aβ signaling pathways, potentially influencing the pathogenesis of AD and playing a significant role in its molecular regulation. Nutritional therapies and probiotic supplements may emerge as a potential treatment strategy to halt the progression of AD. Mossad et al. [45] indicated that the microbially derived metabolite δ-valerobetaine directly influences learning and memory capabilities, with elevated levels observed in elderly mice and humans. Following FMT from young donor mice, there was a reduction in this metabolite’s levels in aged mice. Moreover, metabolites produced by the GMB, which are linked to age-related alterations in the microbiota and are found in higher concentrations in elderly individuals, indicate that certain microbial metabolites may hinder cognitive function in aging populations. In young mice, the administration of isoamylamine led to the death of microglia cells, subsequently resulting in cognitive impairment [31]. The administration of N6-carboxymethyllysine resulted in oxidative stress and mitochondrial damage in the microglia of both young and aged mice. The effect was more pronounced in young mice when the drug was administered intraperitoneally compared to orally, whereas elderly mice exhibited no significant difference in the effects of the delivery method. The results indicate that the intestinal barrier plays a protective role in reducing damage from harmful metabolites derived from microbes associated with aging [46]. It remains unclear whether age-related changes in the gut have positive or negative effects on cognitive function as individuals’ age. Wilmanski et al. [47] found that a lower GMB uniqueness score was associated with increased mortality, whereas a higher score was linked to various health indicators in older adults. Alterations in the gut associated with aging may enhance microbiome diversity, potentially mitigating the effects of aging. Thus, it is plausible that certain probiotic bacteria are increased during healthy aging, while the inability of these bacteria to survive and flourish in an unhealthy aging gut may be attributed to the aging process.

## 3. Fecal Microbiota Transplantation Therapy

This novel therapeutic method entails the transfer of bacteria and metabolites from a healthy donor’s stool sample to a recipient [48]. This method is presently employed for the treatment of *Clostridium difficile* infections, which is classified as a hospital-acquired condition, and is used in conjunction with antibiotic therapy [48]. Healthy microbiota can replicate via FMT, resulting in the production of bioactive metabolites. FMT employs endoscopies, enemas, and the oral feeding of freeze-dried material. Research has examined the efficacy of this method in addressing neurological disorders, including PD, autism spectrum disorder (ASD), and MS [49,50,51,52]. One benefit of this approach is that, even in high-risk individuals, no notable adverse effects have been documented, making it safe. Further research is necessary to completely investigate the potential beneficial and/or negative effects FMT on the aging brain and to fully understand how FMT may affect various areas of overall health in older adults. This approach has the advantage of demonstrating no significant adverse effects in high-risk individuals, indicating its safety. Additional research is required to thoroughly examine the potential positive and negative impacts of FMT on the aging brain and to gain a comprehensive understanding of how FMT may influence different aspects of overall health in older adults. In addition to complete microbiota transfer, attempts have been undertaken to alter AD pathophysiology through the administration of specific microorganisms associated with the disease. Cox et al. [53] reported that weekly treatment with *Bacteroides fragilis* in APP/PS1 mice aged two to five months increased the amyloid plaque burden. Elevated levels of *Bacteroides* were noted in aging Tg2576 transgenic mice and correlated with the presence of amyloid plaques in their brains. Furthermore, preclinical evidence from studies administering antibiotics to rodent models of AD supports the hypothesis that the GMB contributes to the development of the disease. The amyloid plaque load observed in older male mice, as reported by Guil-herme et al. [54]; Kaur et al. [55]; Mezö et al. [10]; and Minter et al. [5], was reduced through the administration of a combination of antibiotics starting at an early age to three distinct amyloid mouse models of AD (APP/PS1, 5xFAD, and AppNL-G-F). No differences were observed in female mice. Furthermore, early administration of an antibiotic cocktail prior to weaning resulted in significant, enduring alterations in the gut microbiome and a reduction in amyloid pathology in later life [10]. Mice exposed to antibiotics during infancy exhibited lasting alterations in peripheral and central immunity, characterized by an increased population of regulatory T cells. Additionally, microglia played a vital role in initiating this response; mice lacking microglia did not exhibit decreased plaque levels following treatment with the antibiotic cocktail, although the removal of microglia did result in a slight reduction in the plaque burden [7]. Mezö et al. [10] demonstrated that the administration of antibiotics to the 5xFAD mouse model of AD led to improved spatial memory and recognition memory performance, a finding also observed in GF mice. In wild-type mice subjected to intracerebral injection of amyloid peptides to simulate the onset of AD at 80 days of age, antibiotic treatment initiated at weaning also resulted in a reduction of anxiety levels [56]. This growing body of evidence connects the GMB to the pathophysiology of AD, indicating its potential as a target for novel therapeutic approaches.

## 4. Strategies for Targeting the Gut Microbiome for Better Brain Health During Aging

**Figure 4:**
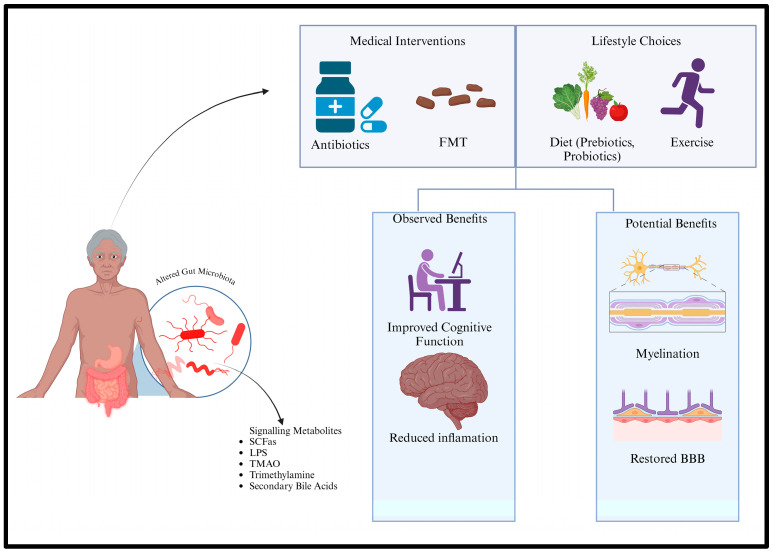
Strategies to alter the GMB for treating neurodegenerative illnesses encompass dietary modifications, prebiotics, probiotics, synbiotics, and fecal microbiota transplantation (FMT). These approaches alter microbial populations and generate compounds such as neurotransmitters and short-chain fatty acids, facilitating neuroprotective effects. Created with BioRender.com.

Investigating the potential of GMB-targeted treatments to mitigate the effects of aging on the brain is essential, given the increasing clarity of the relationship between host brain aging and bacterial influence. The potential for achieving eternal youth in the brain may be associated with various approaches to modifying GMB. These include pharmaceutical interventions such as FMT and antibiotics, alongside dietary and lifestyle modifications like the incorporation of probiotics and prebiotics, adherence to the Mediterranean diet, intermittent fasting, and regular exercise (Figure 4). The subsequent section reviews the literature, highlighting clinical findings, and presenting supporting preclinical data. In addition to its investigation for various other illnesses and disorders, including those impacting brain health, fecal microbiota transplantation is commonly employed as a medical treatment for patients with antibiotic-resistant *Clostridium difficile* [57].

Small-scale clinical trials have shown that FMT can alleviate PD symptoms in patients [58,59], however, the sample sizes in these studies were limited. FMT has been shown to effectively improve mobility metrics in a patient with MS [58,60] and to enhance cognitive performance in two patients with AD who underwent FMT for *Clostridium difficile* infections [60,61]. The findings are corroborated by specific case studies. This indicates that FMT may serve as a potential therapeutic intervention for individuals experiencing neurological disorders related to aging.

The composition of the microbiome is influenced by diet, which subsequently alters the functioning of the GBA. Various treatment strategies have been implemented to address gut microbiome dysbiosis, aiming to restore intestinal microflora balance and enhance clinical outcomes in neurological disorders, including the application of probiotics [62].

The World Health Organization defines “probiotic” as live microorganisms that, when ingested in suitable amounts, can improve host health. The term was first employed in 1974. Probiotics are becoming more popular as dietary supplements and common food products. Probiotics primarily comprise *Bifidobacterium* and lactic acid bacteria, including *Lactobacillus*. Research indicates that metabolites generated by probiotics play a crucial role in mediating host-microbe interactions influenced by dietary factors. Additionally, several gut-resident bacterial species, including *Lactobacillus*, *Ruminococcus*, *Bacteroides*, *Clostridium*, *Bifidobacterium*, and *Peptostreptococcus*, have been identified as capable of synthesizing various tryptophan catabolites, such as tryptamine, 3-methylindole, indoleacetic acid (IAA), and indole [63,64,65].

Recent studies demonstrate that tryptophan catabolites produced by the microbiome affect host health. These metabolites bind to the aryl hydrocarbon receptor (AhR), activating the immune system, improving intestinal barrier function, increasing gastric motility, and enhancing gastrointestinal hormone secretion. Additionally, they exhibit systemic or local antioxidant and anti-inflammatory effects and may influence the gut microbiome and metabolome [66,67]. Tryptophan catabolites produced by commensal microbiota have been shown to activate microglial AhR, thereby inhibiting NF-κB signaling activation and the synthesis of VEGF receptor-β and transforming growth factor alpha (TGF-α) [68]. Dendritic cells display increased AhR levels, which regulate their development and function [69]. Recent studies indicate that probiotics and prebiotics are increasingly employed to modify the gastrointestinal microbiota, with clinical evidence linking these interventions to various neurological disorders, including Parkinson’s disease and ASD. These findings have prompted researchers to investigate the effects of probiotics on different neurological disorders [70].

Numerous studies employing rodent models have demonstrated that probiotic treatment can improve cognitive outcomes in various neurological disorders, such as ASD, epilepsy, and AD. However, the efficacy of probiotic therapy for neurological dysfunction in humans remains inadequately substantiated by clinical studies [57] (Figure 4). They have demonstrated potential in the treatment of human neurodegenerative disorders, including AD. Research indicates that in mouse models of AD, *Lactobacillus plantarum* elevated brain levels of acetylcholine esterase and improved cognitive function [71]. Aβ injection combined with *Lactobacillus acidophilus*, *Lactobacillus fermentum*, *Bifidobacterium lactis*, and *Bifidobacterium longum* in rodent models of sporadic AD has yielded comparable results in other studies [72,73,74].

Prebiotics serve as an alternative to probiotics by modulating gut microbial flora [75]. The International Scientific Association for Probiotics and Prebiotics (ISAPP) characterizes prebiotics as nonviable food components that are selectively utilized by host microbial populations, providing health benefits. This category of chemicals, which includes soluble and fermentable fibers, human milk oligosaccharides (HMOs), and nondigestible oligosaccharides (NDOs), is defined by their capacity to affect gastrointestinal tract health [75] (Figure 4). Few studies have investigated the positive impacts of these compounds on gut microflora in both humans and animals, despite the potential of prebiotic therapy to promote beneficial bacteria like *Lactobacilli* and *Bifidobacteria*.

A recent study demonstrated that prebiotic lactulose may enhance cognitive deficits in an AD mouse model through the stimulation of autophagy and anti-inflammatory processes [76]. These findings indicate that probiotics and prebiotics could be advantageous in the management of neurological disorders. Correlation does not imply causation; thus, additional research is necessary to comprehensively understand the underlying mechanisms.

Synbiotics refer to the integration of prebiotics and probiotics, wherein prebiotics enhance the growth, metabolism, viability, and advantages of probiotic bacteria. This improves the host by augmenting the population of beneficial bacteria in the gastrointestinal tract. The combination used in synbiotics must be suitable to guarantee the survival of probiotic microorganisms within the gastrointestinal tract. Research indicates that the combination of synbiotics with probiotics or prebiotics provides enhanced benefits compared to their individual consumption [77,78].

However, concerns about safety, logistics, and feasibility remain with clinical FMT, particularly in populations where immunodeficiency and fragility are common [79,80]. Therefore, it is essential to develop alternative methods that are less invasive and less harmful for modifying the microbiome–GBA relationship, allowing clinicians to move beyond FMT, even though preclinical studies suggest there are potential benefits of using FMT for brain health in aging.

The use of antibiotics to treat medical conditions is prevalent, with a significant increase in their application among the elderly population. Therefore, it is crucial to determine whether the administration of antibiotics in older adults yields unforeseen positive or negative outcomes. Antibiotics can lead to unforeseen consequences by negatively impacting beneficial microorganisms or promoting antibiotic resistance [80].

In contrast, although information is limited, antibiotics that eliminate a detrimental gut bacterium may enhance cognitive performance. Furthermore, various oral antibiotics can bypass the microbiota–GBA link, exerting direct effects on other systems, including the brain. This is particularly significant in disease states marked by impaired barrier function [81]. Environmental factors, especially air pollution, can significantly influence neurodegeneration through the microbiome–gut–brain axis. Research highlights several mechanisms by which this interaction occurs. Air pollutants, particularly particulate matter, trigger oxidative stress and neuroinflammatory responses. Fine particles can enter the body through inhalation and reach the brain via the olfactory bulb or by crossing the BBB. This process increases the production of pro-inflammatory cytokines like tumor necrosis factor-α (TNF-α) and interleukins (IL-6, IL-1β) in the brain, which are linked to neurodegenerative diseases such as AD and PD [30,82]. Airborne pollutants can alter the composition and diversity of the GMB, impacting the production of metabolites essential for brain health. Changes in gut microbial communities influence systemic inflammation and the neuroinflammatory pathways, potentially exacerbating neurodegenerative processes [83]. Exposure to vehicle exhaust emissions has been shown to disrupt BBB integrity, increasing permeability and allowing harmful substances to enter the brain. This can accelerate neurodegenerative processes, such as the accumulation of Aβ and tau proteins associated with AD [82]. Air pollutants can affect the GBA via the vagus nerve, a critical communication channel between the gut and brain. Dysbiosis caused by environmental toxins can lead to altered signaling through this pathway, contributing to neurological dysfunction [84].

## 5. Conclusions and Perspectives

The GBA represents a complex area of research focused on the interactions between the GMB and the CNS. The precise quantity of metabolites that directly affect the brain after crossing the BBB is unclear, as are the mechanisms of these effects, whether via neuronal or hormonal pathways. Microbial metabolites can directly affect the enteric nervous system and other peripheral nervous system pathways, altering the communication between the peripheral and the central nervous system. Understanding the mechanisms of action in this emerging field poses challenges due to the complexities associated with human neurological disorders and the limitations of using animal models to simulate human diseases. Results from preclinical studies involving specific microorganisms have shown reliability and reproducibility in model systems and laboratories, suggesting a potential for effective application in humans. The integration of microbiology and neuroscience is crucial for developing thorough approaches to clarify the mechanisms underlying these observed results. A comprehensive understanding of classical brain disorders as systemic conditions, particularly with substantial engagement of the gastrointestinal system, could enhance the development of innovative, safe, and effective therapeutic approaches for GMB. Technological advancements such as high-resolution next-generation sequencing, metabolomics, transcriptomics, proteomics, and machine learning are essential for improving our understanding of the microbiota–GBA link in relation to healthy brain aging. Comprehensive demographic studies, mechanistic analyses, and well-designed clinical trials are crucial for improving our understanding of the impact of microbiota-targeted therapies on brain health maintenance in aging populations. Probiotics have shown safety in multiple studies; however, additional research is required to determine any potential harmful effects. Further clinical trials are required to understand the effects of microbial therapeutic interventions, possible combinations with other treatments, suitable sample sizes, and extended follow-up studies. Determining the optimal single or microbial formulation for each specific neurological disorder is essential, as therapeutic outcomes depend on the particular bacterial strain employed. The administration of probiotics and other microbial therapeutics requires careful testing and donor screening to reduce safety risks, especially in the context of the COVID-19 pandemic. Therapies aimed at microbiota may prove advantageous due to the variations in individual gastrointestinal microbiota. Additional research is necessary to fully understand the impact of internal and environmental factors that hinder dietary or other interventions from altering the GMB. Environmental factors, especially air pollution, can significantly influence neurodegeneration through the microbiome–gut–brain axis. Research highlights several mechanisms by which this interaction occurs; metagenomic analysis and multi-omics approaches are crucial for understanding microbial composition at the strain level. Understanding the significance of neuroregulatory systems necessitates an extension beyond the bacteriome. The integration of multi-omics data requires the application of systems biology approaches. Understanding the roles of microbial products and the potential host interactions is crucial, as is elucidating the molecular mechanisms underlying the bidirectional communication between the microbiota and the GBA. Validating the impact of dietary components and microbial metabolites on host physiology and health is crucial for therapeutic strategies. The GMB plays a crucial role in the development of CNS disorders and can be regarded as a distinct organ, often referred to as the “second brain”. Future research in neurotherapeutics is expected to yield important insights regarding the GMB as a potential novel indicator of human health and disease.

## Data Availability

No data was used for the study.
